# Study on Triaxial Properties of Calcareous Sand Modified with Volcanic Ash Cement and Graphene Oxide

**DOI:** 10.3390/ma18174207

**Published:** 2025-09-08

**Authors:** Jun Hu, Zhaokui Sun, Chenming Xu, Zetian Li, Yahui Zhan, Yu Li, Shuai Zhang, Yuxuan Zhou

**Affiliations:** 1 School of Civil Engineering and Architecture, Hainan University, Haikou 570228, China; hj7140477@hainanu.edu.cn (J.H.);; 2 Collaborative Innovation Center of Marine Science and Technology, Hainan University, Haikou 570228, China; 3 School of Information and Communication Engineering, Hainan University, Haikou 570228, China; 4 College of Construction Engineering, Jilin University, Changchun 130026, China

**Keywords:** calcareous sand, volcanic ash cement, graphene oxide, triaxial test, numerical simulation

## Abstract

Calcareous sand, characterized by numerous pore spaces, easy fragmentation, and low strength, is commonly used as fill material in island construction projects. Due to these limitations, it often fails to meet the requirements of actual engineering applications. This paper uses oxidized graphene in combination with fly ash cement to modify calcareous sand. The effects of oxidized graphene, fly ash cement, and curing time on the modification effect were investigated through triaxial tests and numerical simulations. The experimental results show the following: (1) Both the extension of curing age and the increase in the dosage of fly ash cement can improve the shear performance of calcareous sand, with the increase in the dosage of fly ash cement able to ensure thorough bonding between calcareous sand particles. (2) Graphene oxide can significantly improve the shear performance of calcareous sand cement mortar, with the optimal dosage being 0.06%. Excess amounts result in a reduced performance improvement, which is related to the degree of the catalysis of oxidized graphene on hydration reactions. (3) The numerical simulation shows that when the maximum shear stress reached 3437 kPa, cracks began appearing on the specimen, consistent with the experimental results. Meanwhile, the numerical simulation results reveal the crack propagation pattern in the specimens, showing that the stress at crack initiation is lower than the peak stress.

## 1. Introduction

Calcareous sand is a deposit of biological sediments and the remains of marine organisms, primarily composed of calcium carbonate and having a lower hardness than quartz. It is widely distributed in shallow seas, warm seas, and continental shelves. Calcareous sand is characterized by high fragmentation, complex particle shapes, low individual particle strength, large internal voids, and a complex microstructure. Calcareous sand layers are prone to excessive deformation under high loads due to particle fragmentation and sliding [1]. In marine engineering, calcareous sands are commonly used as foundation materials for structures and breakwaters and backfill materials for road embankments or airport runways [2]. The particle structure of calcareous sand has a high porosity (typically between 0.7 and 2.5) due to the presence of a primary bioskeleton, which makes it highly compressible. Engineering data indicates that there is a larger risk of engineering mishaps when the amount of calcium carbonate is greater than 50% [3,4]. Since calcareous sand has special mechanical properties and is the main material for land reclamation, many scholars have taken calcareous sand as an important research object for island engineering. Coop and others [5] showed that particle fragmentation plays a key role in the compressive properties of calcareous sands when the applied pressure exceeds a certain critical value. Salem et al. [6] investigated the effect of the magnitude of perimeter pressure on the mechanical properties of calcareous sands under static loading and cyclic loading by studying two samples with different densities. Zhu et al. [7] revealed the structural characteristics of the internal porosity of calcareous sand particles through microscopic experiments, using MATLAB software for processing and analysis. The distribution of porosity indicates that there are fewer macroscopic pores in the sand and more microscopic pores. Valdes [8] used a single shear instrument to conduct tests on a mixture of calcareous sand and quartz sand, finding that the factors affecting its mechanical properties change with high or low confining pressure.

Currently, numerous researchers have conducted in-depth studies on the issue of modifying and reinforcing calcareous sands. Research by Liu et al. [9] shows that by activating and enriching urease-producing bacteria through biostimulation technology, the precipitation of calcium carbonate in calcareous sands can be promoted, thereby enhancing its mechanical properties. Research by Shen et al. [10] found that MICP (Microbially Induced Carbonate Precipitation) treatment effectively treated individual calcareous sand particles by producing a calcium carbonate coating around the sand particles and improved the particles’ fracture strength, reducing the variability of the particles in calcareous sand. At the same time, the DEM (Digital Elevation Model) modeling technique proposed by them was able to establish an accurate DEM with a real microstructure, particle shape, internal porosity, and the calcium carbonate cement pattern caused by MICP treatment. Liu et al. [11] used a scanning electron microscope (SEM) to discover that microbial bonding treatment can encapsulate calcareous sand particles, improving their bonding and shear strength. Zhang et al. [12] conducted a series of shake table tests to evaluate the seismic performance of calcareous sand foundations treated with MICP. During the vibration process, the excess pore water pressures and surface settlements of the MICP-treated models were significantly reduced. The test results showed that using the MICP method can significantly improve the strength, stiffness, and liquefaction resistance of calcareous sand.

Academics both domestically and internationally have focused on research into the stabilization and modification of materials, with a particular emphasis on microbial reinforcement techniques. However, cement-based composite materials and nanomaterial reinforcement are also commonly used methods. Wang et al. [3] used nano-clay and cement to improve calcareous sand, and the improvement using nano-clay and cement can significantly enhance the strength, stiffness, and liquefaction resistance of calcareous sand. Chai et al. [13] adopted the cement stabilization method to reinforce calcareous sand foundations and studied the basic engineering characteristics, bearing capacity, and deformation behavior of cement-stabilized calcareous sands through a series of experiments. The conclusion drawn was that the cement stabilization method can significantly improve the bearing capacity of calcareous sand foundations. Gao et al. [14] explored the addition of nano-magnesium oxide in soft soils, with research indicating that NM can increase soil particle bonding, fill pores, and enhance water absorption, which significantly enhances the stabilization effect. The autogenous shrinkage of cementitious materials is a difficult problem to solve. Chen et al. [15] studied the effects of nano-magnesium oxide content on the permeability, crack resistance, sulfate corrosion resistance, and freeze–thaw resistance of cement mortar through experiments. The results showed that nano-magnesium oxide has a positive effect on improving the durability of mortar. At the same time, Chen [16] demonstrated through Fourier transform infrared spectroscopy and scanning electron microscopy that the expansion effect of nano-magnesium oxide makes the microstructure of cement-based materials denser and more uniform. Zhang et al. [17] showed that nanometer titanium dioxide enhances the compressive strength of cement mortar by accelerating the hydration of cement and refining the pore structure. Meanwhile, numerous researchers have conducted experimental studies to develop higher-performance cementitious materials. Miao et al. [18] investigated the effects of two industrial byproducts—fly ash (FA) and quartz powder (QZ)—as supplementary cementitious materials (SCMs) on both the macro-properties and microstructure of cement-based materials. The results demonstrated that using these SCMs could significantly reduce CO_2_ emissions and energy consumption. Golewski [19] proposed combining the most commonly used mineral additive (fly ash, FA) with nano-silica (NS) as partial substitutes for ordinary Portland cement (OPC) binder. This approach not only enhanced the concrete’s strength but also reduced OPC demand, consequently lowering CO_2_ emissions.

Graphene oxide is a derivative of graphene, which has a similar structure. Due to its strong van der Waals forces, graphene has hydrophobic properties and is prone to aggregation, which limits its wide application. The emergence of graphene oxide has precisely solved the aforementioned issues [20]. Tong et al. [21] found that the addition of small amounts of graphene nanoparticles (GNPs) and graphene oxide nanoparticles (GONPs) can significantly increase the compressive strength of cementitious materials. The size and functional groups of the nanoparticles have a significant effect on the strength enhancement. The corrosion resistance of cementitious materials can also be improved. Wang et al. [22] found that adding 0.05% graphene oxide to Portland cement paste can reduce the fluidity, increase the viscosity, and shorten the setting time.

As can be seen from the above research, studying the modifications of calcareous sand using volcanic ash cement combined with graphene oxide has a very high degree of feasibility, but there are relatively few related studies. This study uses a graphene oxide composite volcanic ash silicate cement (referred to as volcanic ash cement) as a modifier to solidify and modify calcareous sand. By combining systematic triaxial shear tests and numerical simulations, it deeply explores the influence patterns and mechanisms of graphene oxide and volcanic ash cement composites on the triaxial compression characteristics of modified calcareous sand. The focus is on revealing the evolution characteristics of stress–strain behavior, changes in strength parameters, and shear failure modes of calcareous sand specimens under the synergistic effect of graphene oxide and volcanic ash cement, and clarifying the mechanism by which the composite material system enhances the shear resistance of calcareous sand.

## 2. Test Materials

### 2.1. Calcareous Sand

**Figure 1:**
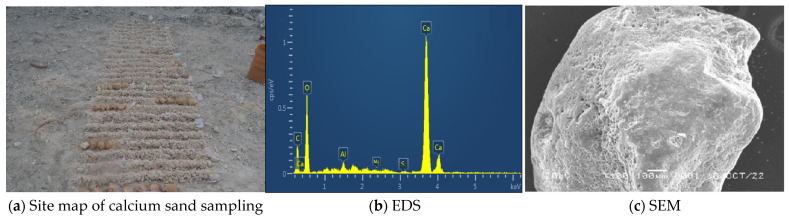
Calcareous sand sampling sites and elemental compositions.

**Figure 2:**
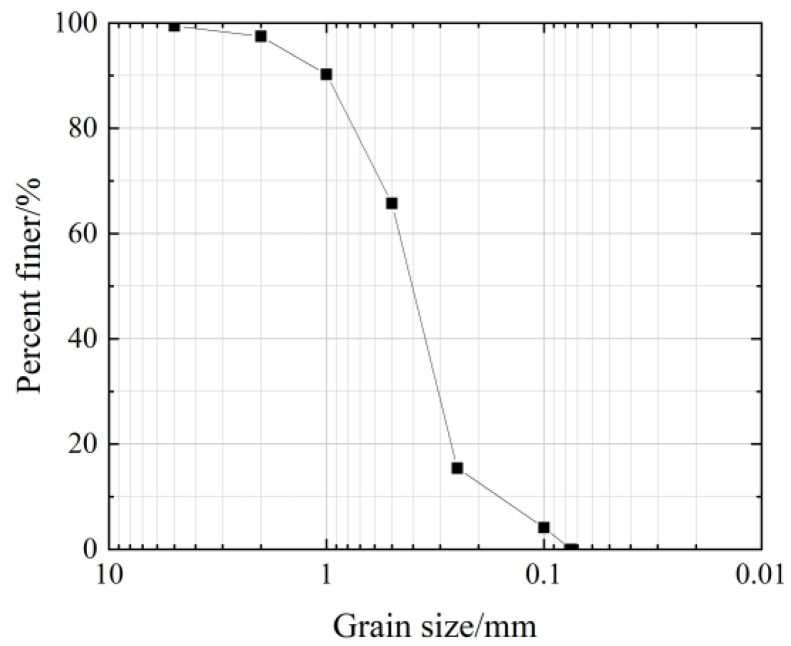
Grain grading curve of calcareous sand.

This experiment’s calcareous sand came from Sansha City in the province of Hainan. Figure 1 shows the sampling site, where calcareous sand grains are loose and uncemented, with a slightly white color, and the sand sample contains a small amount of shell and coral debris. The gradation of the particles is shown in Figure 2, from which it can be seen that *D*_60_ = 0.47, *D*_30_ = 0.32, and *D*_10_ = 0.18. And it can be calculated that the coefficient of uniformity, *C_U_* = 2.61, and the coefficient of curvature, *C_C_* = 1.21.

(1) $$C_{C}=\frac{D_{30}^{2}}{D_{60}\times D_{10}}$$

(2) $$C_{U}=\frac{D_{60}}{D_{10}}$$

### 2.2. Volcanic Ash Cement

**Figure 3:**
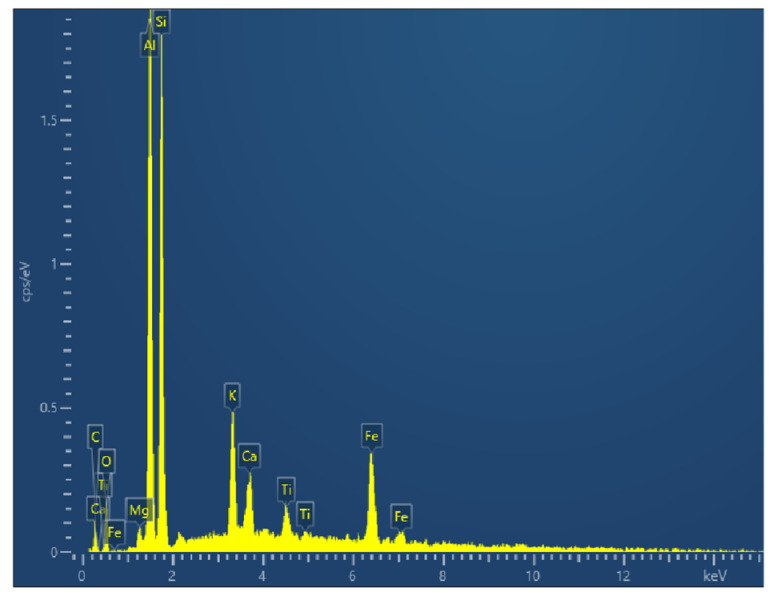
Energy spectrum analysis of volcanic ash cement.

The volcanic ash cement used in the experiment was produced by Zhucheng Qiji Building Materials Co., Ltd., Zhucheng, China. The model of the energy spectrum analysis instrument used in the experiment is Tescan Mira4. The elemental composition of volcanic ash cement was analyzed using EDS, with the specific results shown in Figure 3. The main elements in volcanic ash cement are Si, Al, and Fe, and these elements are mainly present in the form of compounds such as SiO_2_, Al_2_O_3_, and Fe_2_O_3_. The content of SiO_2_ in volcanic ash cement is the highest, and it has a significant impact on the modification effect.

### 2.3. Graphene Oxide

**Figure 4:**
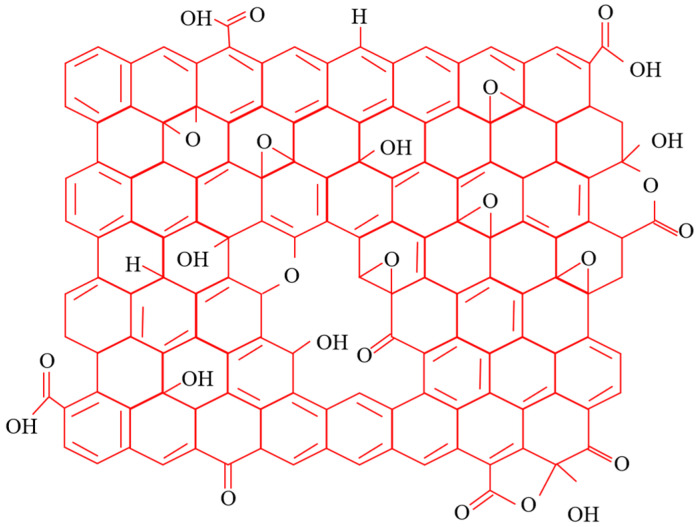
Structure of graphene oxide.

The oxidized graphene used in the experiment was produced by Turing Artificial Intelligence Black Technology Co., Ltd. in Shenzhen, China. Oxidized graphene is a derivative of graphene, having a similar structure but with oxygen functional groups on its surface and edges. Present day GO chemistry is largely inherited from Brodie’s pioneering work on graphite oxide in the 18th century. Graphene is oxidized to obtain graphite oxide. Solution-dispersible, substochiometrically oxidized graphene sheets called GO can be derived from the chemical exfoliation of graphite oxide. Figure 4 presents the graphene oxide structure based on the Lerf–Klinowski model, whose structural formula consists of numerous benzene rings functionalized with oxygen-containing groups [23]. The oxygen functional groups give the oxidized graphene a negatively charged surface, which attracts cations into the layers, expanding the layer spacing and creating conditions for the loading of polymers and inorganic nanoparticles [20].

## 3. Triaxial Test

The incorporation of graphene oxide can improve the compressive properties of calcareous sand volcanic ash cement mortar, and correspondingly has a positive effect on the shear strength, but the mechanism and effect are not clear [24]. This paper conducts unconsolidated undrained shear (UU) triaxial tests on the specimens to investigate the influence of various factors, such as the amount of modified materials, confining pressure, and curing age, on the shear performance of calcareous sand after modification with volcanic ash cement and graphene. The test was conducted under four different confining pressures. To avoid the influence of accidental factors on the test results, five sets of parallel tests were conducted for each condition.

### 3.1. Specimen Preparation

The amount of volcanic ash cement added was 5%, 10%, and 15%, while the amount of oxidized graphene added was 0%, 0.02%, 0.04%, 0.06%, and 0.1%. The mixture is shown in Table 1.

According to the designed mix proportions, the corresponding masses of calcareous sand, volcanic ash cement, graphene oxide, and water were precisely weighed. The volcanic ash cement and graphene oxide were first added to the mixing pot containing calcareous sand and dry-mixed thoroughly. Subsequently, mechanical mixing was performed using the mixing pot.

After complete mixing, the mortar was transferred into pre-prepared molds in three layers, with each layer compacted using a drop hammer. The specimens were demolded after achieving initial strength and then placed in a standard curing room for the specified curing period.

### 3.2. Laboratory Instruments

**Figure 5:**
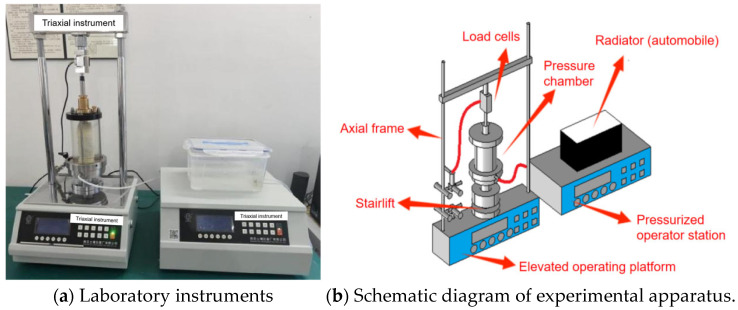
Schematic diagram of the structure of the triaxial test apparatus.

As shown in Figure 5, the triaxial test equipment used in the experiment is a fully automatic triaxial instrument produced by Soil Instrument Factory Co., Ltd. in Nanjing, China, namely the TSZ-2 triaxial instrument. The confining pressure applied during the test ranges from 0 to 5 MPa. The maximum axial load it can withstand is 60 KN, and the shear rate is 0.0001 mm/min to 2.4 mm/min.

### 3.3. The Deviatoric Stress–Strain Curve

**Figure 6:**
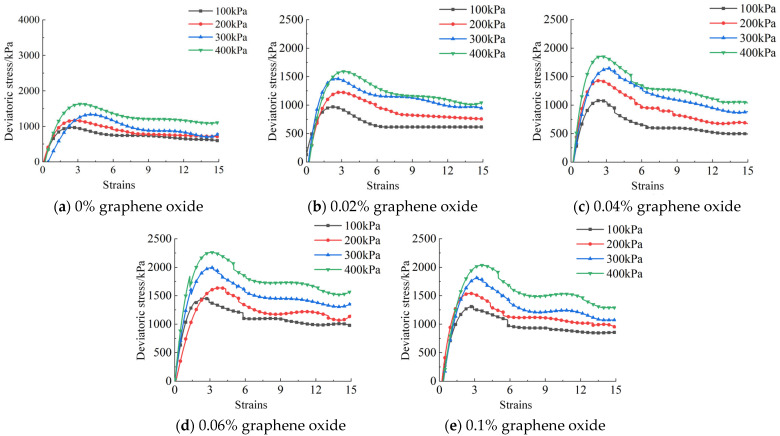
The deviatoric stress–strain curve of the cementitious mortar modified with 10% volcanic ash and graphene oxide after 28 days of maintenance.

A data acquisition system was employed to monitor and record five specimens under each working condition. Using Origin 2024 software, polynomial fitting was applied to process the data from the five specimens under each condition. The fitting yielded a coefficient of determination R^2^ = 0.977, indicating a reasonable fit [24]. They were fitted to obtain the stress–strain curves under the influence of different amounts of modified materials, different confining pressures, and different curing ages. Partial deviatoric stress–strain curves are shown in Figure 6.

The shear performance of the modified specimens was analyzed through the deviatoric stress–strain curves. During the loading process, the specimens gradually began to fail. The deviatoric stress–strain curve mainly shows two stages: (1) During the online elastic deformation stage (axial strain less than 2.8%), there is a significant positive correlation between deviatoric stress and axial strain. In this stage, the cementitious products and the sand grain skeleton jointly bear the load, with no significant relative slippage occurring between particles, and no visible cracks appearing on the specimen surface. (2) When the axial strain reaches the critical range of 2.8–3.2%, a significant peak in deviatoric stress is observed, indicating that the specimen has entered the ultimate bearing state. On the deviatoric stress–strain diagram, this is represented by a curve that suddenly drops and then gradually becomes flat. As the pressure continues to increase, the interaction forces between the particles increase, and large displacements occur between the particles. The specimen begins to fail and cracks appear, and as the number of cracks increases, the specimen gradually loses its bearing capacity.

The peak deviatoric stress is determined by the highest peak point, and the strain corresponding to the peak deviatoric stress is the peak strain. The peak deviatoric stress of the same specimen under high confining pressure is higher than that under low confining pressure. As the confining pressure increases, so does the peak deviatoric stress, and the residual strength also increases. The strain corresponding to the peak deviatoric stress when the specimen reaches the peak deviatoric stress under low confining pressure is less than the strain corresponding to the peak deviatoric stress when it reaches the peak deviatoric stress under high confining pressure. This is because as the confining pressure increases, the specimen enters a high confining pressure state, and the calcareous sand particles break, leading to secondary phase changes during the shear process. At this time, the deviatoric stress–strain curve gradually transitions to a strain-hardening type, and the failure form of the specimen is characterized by shear swelling failure.

When the deviatoric stress is small, the specimen does not produce a significant strain, but as the deviatoric stress increases to a critical state, the material gradually begins to crack and eventually breaks. This is reflected in a sudden drop in deviatoric stress on the deviatoric stress–strain diagram. Finally, as the deviatoric stress gradually increases, the strain slowly decreases. The slope of the deviatoric stress–strain curve and the peak strain vary with different amounts of oxidized graphene, indicating that oxidized graphene has a certain effect on improving the shear performance of calcareous sand, and the improvement ratio also varies. Relevant studies have shown that volcanic ash as a substitute for Portland cement leads to a reduction in the ultimate compressive strength of mortar [25], whereas the addition of an appropriate amount of graphene oxide can greatly increase the compressive strength of calcareous sand specimens.

### 3.4. Mohr’s Circles

To better understand the modification effect of volcanic ash cement and oxidized graphene on the shear strength of calcareous sand, a strength envelope line is drawn based on the Mohr–Coulomb theory and the shear strength parameters C and φ are determined. The strength curve expression is derived and the shear strength expression is given as follows:

(3) $$\tau_{f}=\sigma tan\varphi +C$$

where $\tau_{f}$ is shear strength; C is cohesion; σ is normal stress acting on the shear plane; φ is internal friction angle.

**Figure 7:**
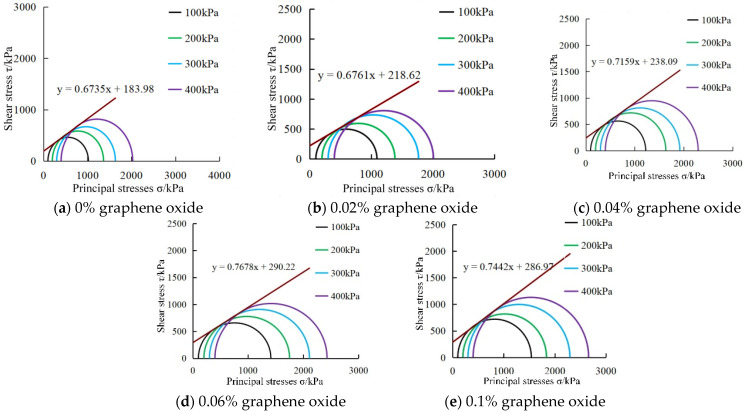
Mohr’s circles for 28-day maintenance of graphene oxide-modified 10% volcanic ash-cemented calcareous sand mortar.

The processing results of some Mohr stress circles are shown in Figure 7. According to the analysis of the Mohr stress circles, as the confining pressure increases, the diameter of the Mohr stress circles also increases, and the strength envelope line is tangential to the Mohr stress circles. The angle of internal friction and cohesion reached a maximum at 0.06% graphene oxide doping. Studies have shown that in undrained triaxial tests, the low saturation state of the specimens leads to a linear increase in strength and confining pressure. It can be seen that the amount of oxidized graphene and the curing age will make the expression of the strength envelope line different. To further explore the impact of the above factors, the content of the analysis is provided in Section 3.5 and Section 3.6.

### 3.5. Analysis of Shear Strength Indicators

#### 3.5.1. The Effect of Volcanic Ash Cement on Shear Strength

The analysis of Figure 7a shows that the numerical value of the cohesion is positively correlated with the amount of volcanic ash cement added and the curing age. When the amount of volcanic ash cement added is 15% and the curing age is 28 days, the numerical value of the cohesion is the highest: 2.55 times that of the cohesion when the amount of volcanic ash cement added is 5% and the curing age is 28 days, and 1.43 times that of the cohesion when the amount of volcanic ash cement added is 15% and the curing age is 7 days. This indicates that the proportion of the increase in cohesion due to the addition of volcanic ash cement is higher than that due to the extension of the curing age. This is because adding more volcanic ash cement fills the pores between calcareous sand particles more compactly and makes the adjacent particles more closely bonded. Extending the curing age enhances the connection strength between adjacent calcareous sand particles, but it does not change the porosity.

The analysis of Figure 7b shows that the height difference in the column chart varies within a small range under different conditions, and the internal friction angle is the largest at a curing age of 28 days. For example, when the amount of volcanic ash cement added is 15% and the curing age is 28 days, the internal friction angle is 1.08 times that of the internal friction angle when the amount of volcanic ash cement added is 5% and the curing age is 28 days, and 1.04 times that of the internal friction angle when the amount of volcanic ash cement added is 10% and the curing age is 28 days.

It can be seen that the amount of volcanic ash cement added and the curing age have an enhancing effect on the internal friction angle and cohesion, with a more significant effect on the cohesion.

#### 3.5.2. The Effect of Graphene Oxide on Shear Strength

**Figure 8:**
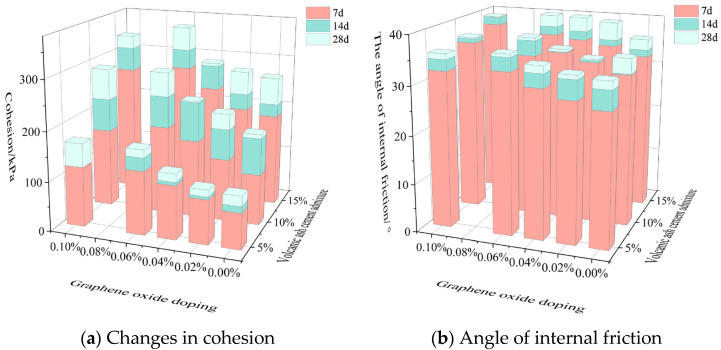
Plot of changes in cohesion and angle of internal friction.

As shown in Figure 8a, when 15% of the volcanic ash cement is used as a binder, the maximum cohesion is achieved when the amount of oxidized graphene added is 0.06% and the curing age is 28 days, with an improvement of 34.45% compared with the case with only 15% of the volcanic ash cement. Similarly, when the amount of oxidized graphene added is 0.02% and the curing age is 28 days, the improvement ratio is 2.85%; when the amount of oxidized graphene added is 0.04% and the curing age is 28 days, the improvement ratio is 7.95%; and when the amount of oxidized graphene added is 0.1% and the curing age is 28 days, the improvement ratio is 24.82%.

As shown in Figure 8b, when 15% of the volcanic ash cement is used as a binder, the maximum internal friction angle is achieved when the amount of oxidized graphene added is 0.06% and the curing age is 28 days, with an improvement of 10.72% compared with the case with only 15% of the volcanic ash cement. Similarly, when the amount of oxidized graphene added is 0.02% and the curing age is 28 days, the improvement ratio is 10.36%; when the amount of oxidized graphene added is 0.04% and the curing age is 28 days, the improvement ratio is 5.36%; and when the amount of oxidized graphene added is 0.1% and the curing age is 28 days, the improvement ratio is 8.02%.

From the above analysis, it can be seen that under the same conditions, the improvement ratio of cohesion is the highest when the amount of oxidized graphene added is 0.06%, and the modification effect is the best. When the amount of oxidized graphene added is increased to 0.1%, the improvement ratio of cohesion shows a decreasing trend.

### 3.6. Analysis of the Peak Shear Stress Indicator

#### 3.6.1. The Effect of Volcanic Ash Cement on Peak Shear Stress

**Figure 9:**
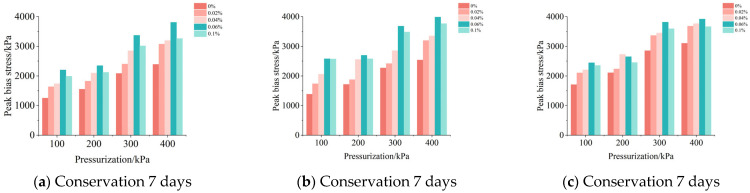
Peak deviatoric force diagram for graphene oxide-modified conditions.

As shown in Figure 9, the content of graphene oxide is 0%. For the same confining pressure, at a confining pressure of 100 kPa, the highest increase in peak stress is 4.17 times; at a confining pressure of 200 kPa, the highest increase in peak stress is 3.55 times; at a confining pressure of 300 kPa, the highest increase in peak stress is 4.00 times; and at a confining pressure of 400 kPa, the highest increase in peak stress is 3.31 times. For the same curing age, at a curing age of 7 days, the highest increase in peak stress is 5.84 times at a curing age of 14 days, the highest increase in peak stress is 5.10 times; and at a curing age of 28 days, the highest increase in peak stress is 4.97 times. For the same amount of volcanic ash cement added, the highest increase in peak stress with 5% of the volcanic ash cement added is 3.09 times; with 10% of the volcanic ash cement added, the highest increase in peak stress is 2.80 times; and with 15% of the volcanic ash cement added, the highest increase in peak stress is 2.48 times.

In summary, the curing age, confining pressure, and amount of volcanic ash cement added all have a positive effect on the peak deviatoric stress of the specimens, but the underlying mechanisms are different. As the curing age increases, the degree of hydration reaction in the specimens gradually becomes more complete, and the peak deviatoric stress of the specimens increases to varying degrees. As the confining pressure increases, the hydration reaction consumes the water in the specimens, causing the specimens in a low saturation state to exhibit isotropic compression and a decrease in porosity, and the strength and stiffness of the modified calcareous sand specimens also increase accordingly. Increasing the amount of volcanic ash cement added can ensure that the volcanic ash cement is fully bonded with the calcareous sand, filling the pores between the calcareous sand particles.

#### 3.6.2. The Effect of Graphene Oxide on Peak Shear Stress

As shown in Figure 9, under the same confining pressure, the maximum enhancement ratio after modification can reach 31.24%. This increase in enhancement ratio is not linear. In some cases, such as under a confining pressure of 400 kPa, the enhancement ratio with 0.04% graphene oxide content is 2.62%, and with 0.1% graphene oxide content is 5.66%, which are both significantly lower than the enhancement ratio of 15.46% with 0.06% content. The same situation exists under other confining pressure conditions, which indicates that there is a threshold value for the content of graphene oxide at which the enhancement effect is most pronounced.

From the analysis above, it is known that the modification effect is best when the content of graphene oxide is 0.06%, and the peak stress enhancement ratio shows a decreasing trend when the content of graphene oxide is 0.1%. However, when the content of graphene oxide is 0.1%, the effect on improving the compressive strength of the volcanic ash cement calcareous sand mortar decreases.

### 3.7. Damage Form

**Figure 10:**
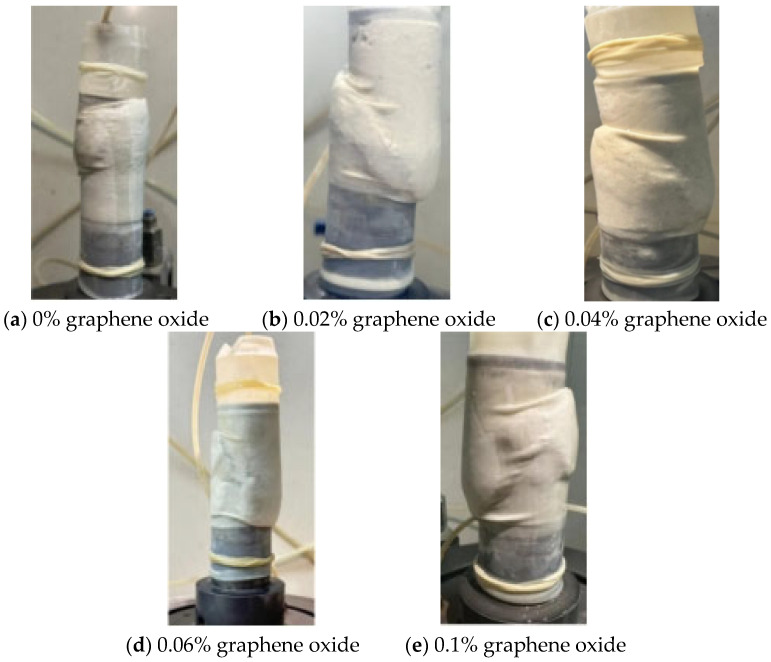
Damage morphology of specimens.

The analysis of the failure morphology of calcium sand specimens with different additions of graphene oxide under load, when the addition of volcanic ash cement and the curing time are the same, is shown in Figure 10. Initially, cracks appear at the top of the cylindrical specimen and then extend to the surrounding area. The outer part of the specimen bulges continuously, and its volume increases. When it reaches a certain “critical state,” the specimen completely loses its load-bearing capacity. Figure 10 shows the failure morphology of the specimen. The cracks start from the outside of the specimen and develop towards the bottom. At this time, the specimen exhibits a clear single-slope fracture. Comparing the failure morphologies of the four specimens with different additions of graphene oxide, it can be seen that the length of the cracks in Figure 10c is greater than those in Figure 10a,b,d. This indicates that under the same load, the path of force transmission in the specimen in Figure 10c is more complete than in Figure 10a,b,d, and its ability to resist the load is stronger. This is because the addition of graphene oxide promotes the hydration reaction between volcanic ash cement and water, enhancing the bonding between calcareous sand particles and volcanic ash cement, thereby reducing internal porosity and allowing the load to be transmitted more limitedly within the specimen.

### 3.8. Mechanism Analysis

In previous studies [24], we observed that tricalcium silicate (Ca_3_SiO_5_) in pozzolanic cement reacts with water to form early hydration products, specifically white Ca(OH)_2_ precipitates. For incompletely reacted pozzolanic cement particles, they tend to adsorb onto the surface of calcareous sand particles. However, as the reaction proceeds more thoroughly, C-S-H gel (3CaO·2SiO_2_·4H_2_O) gradually forms. The chemical reactions are represented by Equations (4) and (5) as follows:

(4) $$2{Ca}_{3}{SiO}_{5}+6H_{2}O\to 3CaO\cdot 2{SiO}_{2}\cdot 4H_{2}O+3Ca{\left(OH\right)}_{2}$$

(5) $$2{Ca}_{3}{SiO}_{4}+4H_{2}O\to 3CaO\cdot 2{SiO}_{2}\cdot 4H_{2}O+Ca{\left(OH\right)}_{2}$$

Without the incorporation of graphene oxide, the hydration reaction of pozzolanic cement remains incomplete. Furthermore, due to the inherently porous structure of calcareous sand, the resulting specimens exhibit excessive porosity, which consequently compromises their overall strength.

The large specific surface area of graphene oxide provides attachment sites for free Ca^2+^ ions, while its oxygen-containing functional groups (-COOH) interact with the C-S-H gel (3CaO·2SiO_2_·4H_2_O) to form a more compact network structure. This enhanced microstructure effectively addresses the porosity issue and improves the mechanical properties of the cementitious composite.

The experimental results demonstrate that graphene oxide exerts a positive effect on the specimen strength, with the maximum strength enhancement achieved at an incorporation content of 0.06%.This is because the hydroxyl (-OH) and carboxyl (-COOH) groups in the added graphene oxide promote the hydration reaction of the volcanic ash cement calcareous sand mortar, thereby making the internal structure more compact. However, when the graphene oxide incorporation content was increased to 0.1%, all strength parameters showed varying degrees of reduction compared with the 0.06% dosage. The main reason is that too many graphene oxide particles cannot be fully unfolded, which in turn weakens the hydration reaction of the volcanic ash cement calcareous sand mortar.

**Figure 11:**
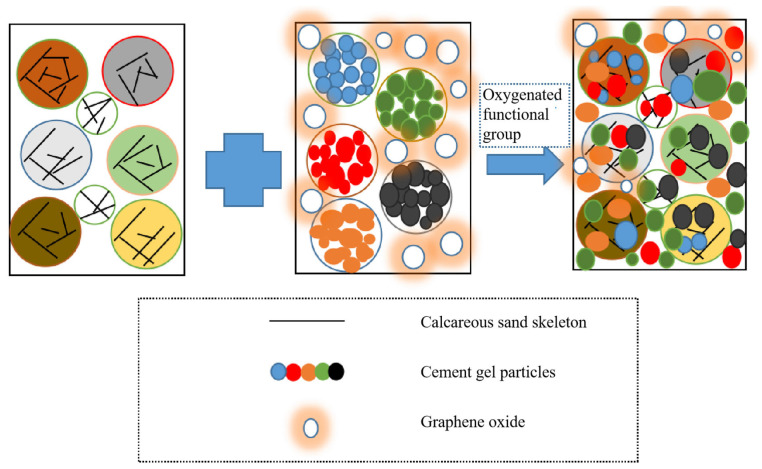
Schematic diagram of the catalytic effect of graphene oxide.

As illustrated in Figure 11, owing to the inherently porous nature of calcareous sand, cement gel particles effectively fill the voids between adjacent calcareous sand particles. The dosage of pozzolanic cement directly determines the quantity of cementitious particles participating in the hydration reaction. Notably, a higher pozzolanic cement content results in stronger interparticle bonding, thereby leading to the further enhancement of the specimen’s mechanical strength.

## 4. Numerical Simulation Study of Triaxial Tests

### 4.1. Modeling and Meshing

Graphene oxide primarily enhances the hydration reaction of pozzolanic cement. Therefore, in the modeling process, constitutive laws for concrete can be adopted as the basis for simulation. Among these, the Concrete Damaged Plasticity (CDP) model in ABAQUS has been extensively utilized for nonlinear analysis.

In the CDP model, concrete failure is primarily characterized by two mechanisms: (1) softening after tensile yielding, leading to cracking; (2) hardening followed by softening after compressive yielding, resulting in crushing. Additionally, the following assumptions must be considered when applying the plastic–damage model: (1) the concrete material is continuum-based; (2) damage in concrete follows an isotropic distribution; (3) the material behavior is predominantly governed by tensile cracking and compressive crushing.

The governing equations of the Concrete Damaged Plasticity model are as follows [26]:

(6) $$\sigma_{t}=(1-d_{t})E_{0}(\varepsilon -{\varepsilon_{t}}^{pl})$$

(7) $$\sigma_{c}=(1-d_{c})E_{0}(\varepsilon -{\varepsilon_{c}}^{pl})$$

where $\sigma_{c}$ is the compressive inelastic stress; $d_{c}$ is the compressive damage variable; ${\varepsilon_{c}}^{pl}$ is the compressive equivalent plastic strain (inelastic); $E_{0}$ is the initial elastic modulus; $\sigma_{t}$ is the tensile inelastic stress; $d_{t}$ is the tensile damage variable; ${\varepsilon_{t}}^{pl}$ is the tensile equivalent plastic strain (inelastic).

When defining the compressive damage behavior, key parameters such as the dilation angle, eccentricity, and *f_b_*_0_/*f_c_*_0_ (where *f_b_*_0_ represents the biaxial compressive strength of concrete and *f_c0_* denotes the uniaxial compressive strength) are configured as presented in Table 2.

The CDP model directly characterizes the material’s mechanical properties through the initial elastic modulus. Based on experimental data, the specific value of the elastic modulus is calculated, and the corresponding Young’s modulus is subsequently determined through conversion relationships. In this case, the parameter is set to 21,248.7.

When defining damage behavior, directly applying the damage evolution parameters provided by design codes to the Concrete Damaged Plasticity (CDP) model may lead to computational convergence difficulties. To determine the damage variables, Equations (6) and (7) are typically transformed, resulting in the derived calculation formulas shown in Equations (8) and (9).

(8) $$d_{c}=1-\sqrt{\frac{\sigma_{c}}{E_{0}\varepsilon_{c}}}$$

(9) $$d_{t}=1-\sqrt{\frac{\sigma_{t}}{E_{0}\varepsilon_{t}}}$$

**Figure 12:**
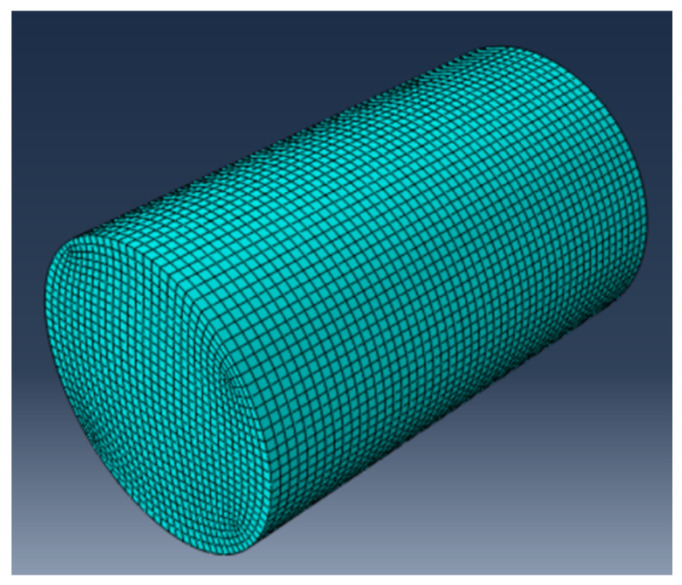
Simulation model of the unconfined test.

The standard cylindrical triaxial test model (diameter 39.1 mm, height 80 mm) was modeled using the ABAQUS 2020 software, employing a plasticity–damage constitutive model. The neutral axis algorithm was used to optimize the mesh division to ensure quality and regularity, as shown in Figure 12. The actual mechanical test data were converted into real stress and strain data and imported into the dataset, and the elastic–plastic material behavior was set up to complete the material mapping.

**Figure 13:**
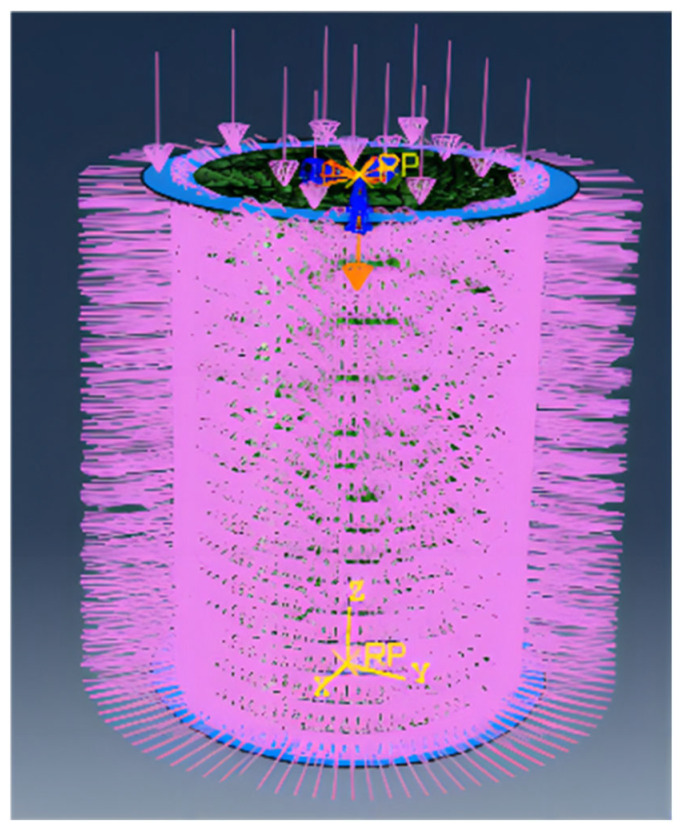
Simulation of sample stress.

In the simulation, boundary conditions were set to simulate the confining pressure action of the specimens in the triaxial test, restricting three-directional displacements but allowing for angular displacement. The simulation was terminated when the strain of the specimen reached 15%. The curve under the condition of 300 kPa confining pressure was selected for study, and the hydraulic pressure acting on the specimen in each direction was indicated by red arrows, as shown in the specific simulation diagram in Figure 13.

### 4.2. Analysis of Numerical Simulation Results

**Figure 14:**
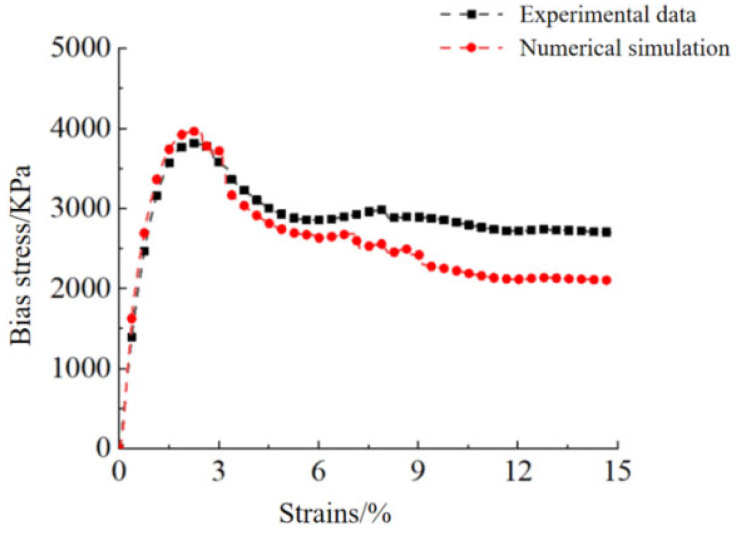
Simulation results of stress–strain curve of triaxial shear test.

As shown in Figure 14, the experimental curve and the numerical simulation result curve have a high degree of agreement in each loading stage. Specifically, from the moment the specimen is subjected to the confining pressure until it reaches the peak stress stage, the two stress–strain curves are well matched. The numerical simulation curve can reflect well the actual experimental data curve. As the confining pressure continues to act on the specimen, it begins to fail, and its load-bearing capacity decreases. During this stage, the “degree of agreement” between the numerical simulation curve and the experimental data curve shows a decreasing trend.

**Figure 15:**
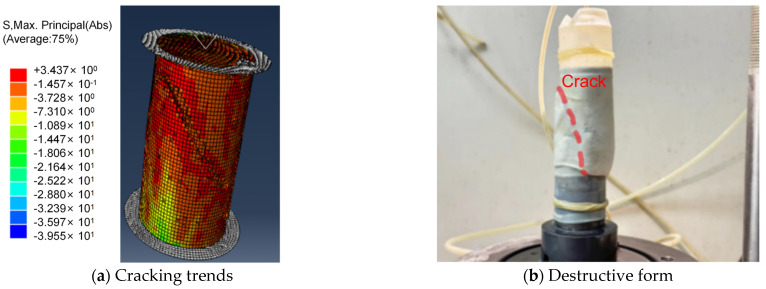
Comparison chart of numerical simulation and experimental research.

The stage of crack formation typically occurs after the peak deviatoric stress is reached; therefore, we also selected to observe the development of cracks under the maximum stress state. As shown in Figure 15a, when the maximum shear stress reached 3437 kPa, noticeable cracks appeared on the surface of the specimen. These cracks started from the top of the specimen and gradually expanded towards the outer side, eventually forming some line segments with a certain inclination angle. The cracks in Figure 15b correspond to the simulation results, indicating that the conclusions obtained from the numerical simulation are consistent with the experimental results, further verifying the accuracy of the simulation outcomes.

In summary, based on the mutual verification of the two parts of the simulation results, it is demonstrated that the parametric modeling conducted under the “plasticity–damage constitutive model” yields simulation results that exhibit a good correlation with the experimental results.

## 5. Conclusions

This paper uses volcanic ash cement and graphene oxide as modifying materials to conduct unconsolidated undrained shear (UU) triaxial tests on specimens to explore the effects of different additions, confining pressures, and curing ages on the shear performance of calcareous sand. Simultaneously, the mechanical tests are numerically simulated using the ABAQUS software. The main work includes fitting the stress–strain curves, simulating the development of cracks, and other studies. The experimental and numerical simulations are mutually verified, leading to the following conclusions:

(1) As the curing age increases, the degree of hydration reaction in the specimens becomes more thorough, resulting in a certain degree of increase in the peak stress of the specimens. The hydration reaction of the cement consumes the water in the specimens, and as the confining pressure increases from 100 kPa to 400 kPa, isotropic compression and a decrease in porosity are observed in the specimens with low saturation, and the strength and stiffness of the modified calcium sand specimens also increase.
(2) The addition of volcanic ash cement can ensure sufficient bonding between volcanic ash cement and calcareous sand, filling the pores between calcareous sand particles. The content of volcanic ash cement significantly affects the shear strength. With the increase in the content of volcanic ash cement, both cohesion and internal friction angle show a positive correlation increase, with the improvement in cohesion being more significant. At a volcanic ash cement content of 15% and a curing age of 28 days, the cohesion reaches its maximum, which is 2.55 times that of a 5% content. In addition, the addition of volcanic ash cement can significantly enhance the peak deviatoric stress, with the highest increase at a 15% content, reaching 2.48 times.
(3) This study demonstrates that graphene oxide plays a crucial role in optimizing the hydration process and microstructural development of pozzolanic cement–calcareous sand systems, leading to superior performance in practical applications. Under the same conditions, the best improvement in cohesion and internal friction angle is achieved with a graphene oxide content of 0.06%, and the peak deviatoric stress is also the highest. Under the conditions of a volcanic ash cement content of 15% and a curing age of 28 days, the optimal content of graphene oxide is 0.06%, at which the improvement in cohesion and internal friction angle is the greatest, at 34.45% and 10.72%, respectively. Moreover, when the content of graphene oxide is increased to 0.1%, the improvement in cohesion and internal friction angle begins to decrease. This is related to the catalytic degree of the hydration reaction of volcanic ash cement by graphene oxide.
(4) Through numerical simulation, the development of cracks in the specimens is simulated. When the axial stress of the specimens reaches its peak, the internal structure begins to fail, and the load-bearing capacity of the specimens starts to decrease. However, the “relative displacement” between particles does not occur immediately, which results in the stress corresponding to the generation of cracks being less than the peak stress. The cracks develop from the top of the specimen to the outer side, and finally, the cracks appear as segments with a certain inclination angle. As the test continues, the cracks develop toward weaker areas. When the crack extends beyond the boundary range, it manifests as a bulging phenomenon during the test process.

## Figures and Tables

**Table 1:** Test mix ratios.

Test No.	Water–Cement Ratio	Volcanic Ash Cement Mixing	Graphene Oxide Doping	Age of Conservation
1	0.6	5%	0%	7 d, 14 d, 28 d
2			0.02%	
3			0.04%	
4			0.06%	
5			0.10%	
6		10%	0%	
7			0.02%	
8			0.04%	
9			0.06%	
10			0.10%	
11		15%	0%	
12			0.02%	
13			0.04%	
14			0.06%	
15			0.10%	

**Table 2:** Parameter setting.

Dilation Angle	Eccentricity	*f_b_*_0_/ *f_c_*_0_	Young’s Modulus	Poisson’s Ratio
30	0.1	1.16	21,248.7	0.2

## Data Availability

The original contributions presented in this study are included in the article. Further inquiries can be directed to the corresponding author.
